# Are diabetes management guidelines applicable in ‘real life’?

**DOI:** 10.1186/1758-5996-4-47

**Published:** 2012-11-21

**Authors:** Luciana V Viana, Cristiane B Leitão, Maria de Fátima Grillo, Ennio P C C Rocha, Juliana K Brenner, Rogério Friedman, Jorge L Gross

**Affiliations:** 1 Endocrine Division and Primary Care Unit, Hospital de Clinicas de Porto Alegre and Federal University of Rio Grande do Sul, Rua Ramiro Barcelos, 2350 – Prédio 12 – 4° andar, Porto Alegre, RS 90035-003, Brazil

**Keywords:** Type 2 diabetes, ADA guidelines, Real life

## Abstract

**Background:**

The American Diabetes Association (ADA) has published several diabetes treatment algorithms, but none have been tested in real-life settings. The aim of this study is to analyze the feasibility of achieving and/or maintaining HbA_1c_ levels <7.0% using current diabetes treatment guidelines and the resources available in the public health care system of Brazil.

**Methods:**

A one-year, single-arm interventional study was conducted with type 2 diabetes patients in a primary care unit. Intervention consisted of intensification of lifestyle changes and sequential prescription of drugs based on ADA guidelines using the medications available through the publicly funded Unified Health System (*Sistema Único de Saúde*, SUS).

**Results:**

Ninety patients (age: 62.7±10.4 years; diabetes duration: 8.2±9.1 years) completed the trial. During the intervention period, increases were observed in number of oral antidiabetic agent (OAD) classes per patient (1.50±0.74 vs. 1.67±0.7; p=0.015), OAD pills per patient (2.64±1.89 vs. 3.33±2.23 pills/patient; p <0.001), insulin dosage (0.20±0.29 vs.0.50±0.36 UI/kg/day; p=0.008) and number of patients on insulin (19 [21%] vs. 31 [34%]; p<0.01), but no improvement in HbA_1c_ (7.2±1.6% vs. 7.3±1.5%; p=0.453) or frequency of patients on target, defined as HbA_1c_ <7% (53.3% vs. 48.9%; p=0.655). Patients with baseline HbA_1c_ <7% had a small increase in HbA_1c_ during the trial (6.3±0.4 vs. 6.7±0.9%; p=0.002). No such change was observed in those with baseline HbA_1c_ ≥7%.

**Conclusions:**

In this group of patients with a mean baseline HbA_1c_ of 7.2%, implementation of 2006/2009 ADA/EASD guidelines led to achievement of the therapeutic goal of HbA_1c_ <7% in a small proportion of patients.

## Introduction

Both the American Diabetes Association (ADA) and the European Association for the Study of Diabetes (EASD) have published algorithms for management of hyperglycemia in patients with type 2 diabetes [1,2]. According to these algorithms, the first step of diabetes treatment should consist of lifestyle intervention plus metformin. If optimal glycemic control is not achieved, step two consists of addition of either a sulphonylurea or basal insulin. These recommendations have not, however, been tested in real-life settings. In Brazil, most patients with type 2 diabetes are treated at primary care clinics and have access to metformin, sulfonylureas, and NPH insulin, which are provided free of charge by the public health care system, the Unified Health System (*Sistema Único de Saúde*, SUS). Therefore, the aim of this study was to analyze whether an HbA_1c_ level of <7.0% can be achieved and maintained in patients with type 2 diabetes treated at a primary care clinic in accordance with ADA/EASD guidelines.

## Research design and methods

### Study design and setting

This one-year, open-label, uncontrolled, single-arm interventional study was conducted at a primary care clinic located in the metropolitan area of the city of Porto Alegre. This clinic is managed by Hospital de Clínicas de Porto Alegre, a university hospital and reference center, and is responsible for the care of approximately 40,000 patients.

The study protocol was approved by the Hospital de Clínicas de Porto Alegre Research Ethics Committee and registered in the Clinical Trial Protocol Registration System (ID 06260). All patients provided written informed consent.

### Patients

Consecutive adult (age >18 years) patients with type 2 diabetes who attended the primary care clinic regularly during the 6 months preceding the screening visit were invited to take part in the study. The exclusion criteria were: history of active infection (e.g. osteomyelitis, pulmonary tuberculosis, AIDS); chronic corticosteroid use; unstable angina or myocardial infarction in the last 3 months; advanced renal disease (requiring renal replacement therapy); heart failure (New York Heart Association class III and IV); cirrhosis; alcohol or illicit drug use; dementia; current pregnancy or lactation; and current cancer or any disease that might affect survival during the next 5 years.

### Baseline evaluation

At baseline, patients underwent an evaluation consisting of a standard history and physical examination. Patients were classified as current smokers or nonsmokers. Ethnicity was self-reported as white or nonwhite. Past medical history was evaluated clinically. Microalbuminuria was defined by an albumin level >17 mg/L on a random spot urine sample [3]. Cerebrovascular disease was defined by the presence of a history of stroke and/or findings consistent with sequelae of stroke. Heart disease was defined by a history of myocardial infarction, angina or heart failure and, when available, diagnosed directly by myocardial perfusion scintigraphy and coronary angiography. Body mass index (BMI) was calculated using the formula [weight (kg)/height^2^ (m)].

Blood pressure was measured twice during each visit, with patients in the sitting position and after a 10-minute rest, with an OMRON HEM-720 Automatic Blood Pressure Monitor. Hypertension was defined as blood pressure levels ≥140/90 mmHg or use of antihypertensive drugs.

### Interventions

The study comprised 3 stages: a run-in period (3 months), the drug intervention period (6 months) and the stabilization period (2-3 months), and was conducted by an endocrinologist (LVV) and a generalist nurse (MFG). Eligible patients underwent an interview, clinical examination and laboratory workup (glucose, HbA_1c_ [HPLC], lipid profile, liver function tests, creatinine and spot urine albumin). Lifestyle modification advice was provided in a 1-hour appointment during the first study visit, and a folder containing a diet plan and recommendation of at least 150 minutes of physical exercise per week was given to each patient. During the run-in period, patients received a glucose monitoring device and test strips and given guidance on how to use the device and record measurement results. Patients were asked to carry out fasting blood glucose monitoring (before breakfast), but only three times per week due to economic constraints. Patients returned to the primary care clinic for monthly follow-up and reminders of dietary guidance and the importance of exercise and adherence to current medications. During the intervention period, participants visited the clinic once monthly for weight and blood pressure checks and review of the results of self-monitoring of blood glucose (SMBG). The goal was to achieve fasting capillary blood glucose levels (as measured by SMBG) in the range of 90 to 130 mg/dL. If mean SMGB values were higher than 130 mg/dL, medications were added in the following sequence: metformin; glibenclamide; and NPH insulin, initially at bedtime and, if goals were still not met, before breakfast as well, according to the 2006 Diabetes Treatment Algorithm [1]. Medications were started at the lowest manufacturer-recommended dose and doses were increased to the maximum tolerated level at monthly intervals, as guided by SMBG. Another class of glucose-lowering medication was added after the maximum dose was reached. HbA_1c_ was measured every 3 to 4 months for further adjustment of diabetes medications. The last 2–3 months of the study (stabilization period) were used to observe whether participants’ HbA_1c_ levels had stabilized after the treatment modifications performed during the intervention period. Throughout the study period, patients received standard medical care at the primary care clinic for any adverse events or other concomitant illnesses.

The study endpoints were change in HbA_1c_ after the intervention and the proportion of patients achieving and/or maintaining an HbA_1c_ of < 7% during 1-year follow-up.

### Statistical analysis

Results are expressed as mean ± SD, median (interquartile range) or N (%). Student’s t test, the Mann-Whitney U test or chi-square test were used for comparisons. Multivariate logistical analyses were performed to determine which factors were associated with HbA_1c_ >7% (dependent variable). Independent variables were selected on the basis of their significance on univariate analyses and/or biological relevance. Sample size was calculated considering a 0.5% reduction in HbA_1c_ with 1.5% SD. P values <0.05 (two-sided) were considered statistically significant. All analyses were performed in SPSS 15.0 (Chicago, IL, USA).

## Results

A total of 116 patients agreed to take part in the study, but 26 did not complete the trial: 3 withdrew consent, 16 were lost to follow-up, 2 died, 1 suffered a stroke with significant sequelae, and 4 developed cancer. These participants did not differ from those who completed the trial in terms of age, duration of diabetes, gender distribution, ethnicity, or baseline HbA_1c_. Ninety patients (age: 62.7±10.4 years, women: 57.8%, whites: 78.9%, diabetes duration: 8.2±9.1 years, BMI: 29.8±4.9 kg/m^2^, systolic blood pressure: 144.3±22.7 mmHg) completed the trial (Table 1).

**Table 1:** Baseline clinical and laboratory characteristics of type 2 diabetic patients included in the study

**Baseline**	
N	90
Age (years)	62.7 ± 10.4
White ethnicity	71 (78.9%)
Women	52 (57.8%)
Diabetes duration (years)	8.2 ± 9.1
Primary care unit attendance (years)	2.1 ± 2.5
Previous cardiovascular event	21 (23.3%)
Current Smoking	13 (14.4%)
Hypertension	79 (89.8%)
SBP (mmHg)	144.3 ± 22.7
DBP (mmHg)	79.4 ± 10.7
BMI (kg/m^2^)	29.8 ± 4.9
Using statin	45 (50%)
Using aspirin	55 (61.1%)
Microalbuminuria	20 (23.8%)
Treatment Type	
Diet only	10 (11.1%)
One oral agent	33 (36.6%)
Metformin	30
Glybenclamide	3
Two oral agents	28 (31.1%)
Insulin use	19 (21.1%)
NPH alone	4
NPH + Metformin	14
NPH + Glybenclamide	0
NPH + Metformin + Glybenclamide	1
Total cholesterol (mg/dl)	179.1 ± 41.2
HDL cholesterol (mg/dl)	47.5 ± 11.8
Triglycerides (mg/dl)	153 (109.0 -216.5)
LDL cholesterol (mg/dl)	94.9 ± 33.0
Creatinine (mg/dl)	0.86± 0.24
HbA1c (%)	7.2 ± 1.6

Data are mean ± SD, number of patients with the characteristic (%).

**Figure 1:**
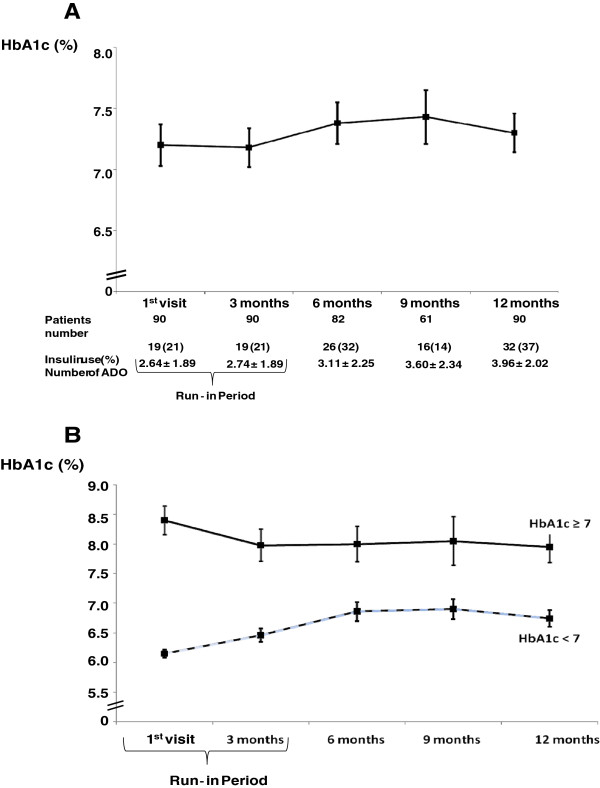
**HbA1c values during the study: Panel A – General view of the HbA1c in the 90 patients and medication prescribed during the study.** Panel B – HbA1c ≥7% and HbA1c <7% behavior throughout the study.

At enrollment, 10 (11%) patients were treated with dietary measures alone, 30 (33%) with metformin alone, 3 (3%) with a sulphonylurea alone, 28 (31%) with metformin and a sulphonylurea combined, and 19 (21%) were on insulin (4 on insulin alone). During the intervention period, the number of oral agents employed rose (1.50±0.74 vs. 1.67±0.7; p=0.015), as did the pill burden (2.64±1.89 vs. 3.33±2.23 pills/patient; p <0.001). Several patients started insulin therapy, increasing the number of patients on insulin from 19 (21%) to 31 (34%) (p <0.01). There was also a significant increase in mean insulin dosage (0.20±0.29 vs.0.50±0.36 UI/kg/day; p=0.008) in patients who had been on insulin since baseline; despite this increase, no episodes of severe hypoglycemia were reported. At baseline, mean HbA_1c_ was 7.2±1.6%, and no change was observed during the follow-up period (7.30±1.48%; p=0.453; Figure 1A). The number of patients with HbA_1c_ within target values was 48 (53.3%) at baseline and 44 (48.9%) at the end of the study (p=0.655). No individual factor could predict final HbA_1c_ ≥7%, except for age at diabetes onset (OR: 0.963; 95%CI 0.930–0.997; p=0.033) and insulin use at baseline (OR: 3.412; 95%CI 1.110–10.491; p=0.032).

Based on the mean of two initial HbA_1c_ measurements (baseline and end of run-in), patients were divided into two groups: HbA_1c_ <7% (n=55, 61%) and HbA_1c_ ≥7% (n=35, 39%). No between-group differences in age, gender, diabetes duration, and BMI were detected. Patients with HbA_1c_ <7% had a significant increase in HbA_1c_ (6.30±0.43 vs. 6.71±0.90%; p=0.002) during the study period, while those with HbA_1c_ ≥7% did not experience such changes (8.6±1.5% vs. 8.3±1.7%; p=0.64) (Figure 1B). At the end of the study, 39 (71%) of 55 patients still has HbA_1c_ levels <7%, whereas only 7 (20%) of 35 patients in the baseline HbA_1c_ ≥7% group reached this goal.

## Conclusions

In this sample of patients with type 2 diabetes attending a primary care clinic, recommendation of lifestyle modifications and intensification of treatment with traditional antihyperglycemic agents were not enough to decrease HbA_1c_ to (or or maintain A_1c_ at) ADA/EASD goals. It is widely recognized that most antidiabetic treatments fail as monotherapy as time goes by [4,5], and that administration of additional antihyperglycemic agents, including insulin, enables achievement of HbA_1c_ goals in approximately 50% of patients [6,7]. In our study, only 16% of patients reached the target of HbA_1c_ <7%, increases in dosage and number of antihyperglycemic agents notwithstanding.

It should be noted that this cohort of patients was relatively well controlled (mean HbA1c 7.2%; 6.1–7.9%), which is far below the expected for DM patients in Brazil. In Brazil, the prevalence of inadequate metabolic control (defined as HbA_1c_ >7%) in the diabetic population is 76% [8], and in the latest countrywide diabetes surveillance study, the median HbA_1c_ of Brazilian type 2 diabetic patients was 8.1% [9]. Extrapolation of data from this study requires caution, as it was conducted at a primary care clinic run by a university hospital. Nevertheless, it shows that good glycemic control can be achieved with the resources available in the public health care system through application of international clinical guidelines.

Baseline HbA1c might be a determinant of glycemic response to antidiabetic therapies [10,11], and a small reduction in HbA_1c_ could be expected in this sample. Even so, a small increase in HbA_1c_ in patients with HbA_1c_ <7% was observed, whereas no improvement was found in those with higher HbA_1c_ levels at baseline. Since diabetes is a progressive disease, stability of HbA_1c_ levels during the study period can also be considered a partial success.

Limitations of this study include the absence of a control group and the small sample size. In a French study of similarly standardized diabetes care, no improvement in A_1c_ was observed in the interventional group over the course of the trial (7.5±1.8 vs. 7.2±1.5; p=0.1), but deterioration occurred in the control group, resulting in a between-group difference of -0.87% at the end of the trial [12]. Recently, the ADA and EASD published a new patient-centered strategy for management of diabetes. This new protocol still uses the same principles applied in this study, but is less centered on HbA_1c_ targets [13]. On the basis of recent evidence [14,15], individualization of HbA_1c_ goals seems reasonable, and less strict glycemic control may be achievable with the medications available in the Brazilian Unified Health System.

In conclusion, implementation of the ADA/EASD 2006/2009 guidelines led to achievement of HbA_1c_ <7% in a small proportion of patients with type 2 diabetes. It bears noting that the included patients had good metabolic control—far beyond that of the general Brazilian diabetic population—at baseline. In this group of patients, review of anti-hyperglycemic management strategies, perhaps employing a more aggressive lifestyle intensification strategy [16] and/or including new classes of antidiabetic agents, could ensure optimal blood glucose control.

## Competing interest

Nothing to declare.

## Authors’ contributions

LVV researched data and drafted the manuscript. MFG, EPPCR and JKB researched data. CBL, RF and JLG reviewed/edited the manuscript and contributed to discussion. All authors read and approved the final manuscript.
